# COVID-19-Pandemiekrisenstäbe: Organisation, Befugnisse und Herausforderungen – Strukturelle Gegebenheiten verstehen und nutzen

**DOI:** 10.1007/s00103-022-03542-x

**Published:** 2022-05-03

**Authors:** Isabell Klinger, Maria Heckel, Sophie Shahda, Ursula Krisen, Silke Stellmacher, Sandra Kurkowski, Christian Junghanß, Christoph Ostgathe

**Affiliations:** 1 Palliativmedizinische Abteilung, Friedrich-Alexander-Universität Erlangen-Nürnberg, Krankenhausstr. 12, 91054 Erlangen, Deutschland; 2 Zentrum für Innere Medizin, Medizinische Klinik III – Hämatologie, Onkologie, Palliativmedizin, Universitätsmedizin Rostock, Rostock, Deutschland

**Keywords:** Deutschland, SARS-CoV‑2, Krisenmanagement, Föderalismus, Gesundheitsversorgung, Germany, SARS-CoV‑2, Pandemic management, Federalism, Healthcare

## Abstract

**Hintergrund und Ziel:**

Im föderalen Deutschland sind Krisenstäbe zentrale Instrumente der Pandemiebewältigung. Das Ziel dieses Artikels ist es, Strukturen und Befugnisse von COVID-19-Pandemiekrisenstäben zu beschreiben, die während einer Studie zur Versorgung Schwerkranker und Sterbender in Pandemiezeiten (PallPan) exploriert wurden. Der Schwerpunkt liegt auf gesundheitsbezogenen Krisenstäben von Bund und Ländern (Makroebene), von Landkreisen, kreisfreien Städten und Kommunen (Mesoebene) sowie auf den Krisenstäben einzelner Einrichtungen der Gesundheitsversorgung (Mikroebene).

**Methoden:**

Die Mitglieder der Krisenstäbe wurden mittels qualitativer semistrukturierter Interviews (10/2020–02/2021) befragt. Die Auswertung erfolgte mittels qualitativ strukturierender Inhaltsanalyse.

**Ergebnisse:**

42 Personen berichteten über 43 Krisenstäbe in 14 Bundesländern. Einheitliche Regelungen hinsichtlich der Initiierung, personellen Zusammenstellung, Aufgaben, Zuständigkeiten und Befugnisse von Krisenstäben gibt es in Deutschland nicht. Auf Makroebene werden rechtliche und finanzielle Voraussetzungen zur Pandemiebewältigung geschaffen. Die Hauptverantwortung für die Umsetzung von Maßnahmen zum Schutz der Gesundheit liegt bei den Krisenstäben der Meso- und Mikroebene selbst. Die Vorgaben der Gesundheitsämter sind dabei maßgeblich für die Krisenstabsarbeit. Zentrale Aufgaben und Maßnahmen bezogen sich auf die Informationsbereitstellung und die Beschaffung und Verteilung von Ressourcen.

**Diskussion:**

Die gewonnenen Erkenntnisse zu Strukturen und Befugnissen von Krisenstäben können Interessenvertretungen dabei helfen, die Anliegen zur Aufrechterhaltung der Gesundheitsversorgung spezifischer Bevölkerungsgruppen, wie beispielsweise schwerkranker und sterbender Menschen, in Pandemiezeiten gezielter zu adressieren.

## Abstract (en)

**Background and aim:**

Germany has a federal state system. Pandemic response teams are key instruments of pandemic management. The aim of this article is to describe the structures and powers of pandemic response teams that were explored during a study on the care of the critically ill and dying in times of a pandemic (PallPan). The focus is on health-related pandemic response teams on the national state level (macrolevel) and federal and community level (mesolevel) as well as pandemic response teams in healthcare facilities (microlevel).

**Methods:**

Members of pandemic response teams took part in qualitative semi-structured interviews (October 2020–February 2021). The evaluation was carried out by means of qualitative structuring content analysis.

**Results:**

Forty-two persons reported on 43 crisis teams from 14 federal states. Response teams in healthcare facilities and public administration differ primarily with regard to their competencies. Officially predetermined regulations regarding the initiation, personal composition, tasks, responsibilities, and competencies of pandemic response teams are not predefined in Germany. The macrolevel defined the legal and financial conditions for pandemic management. Meso- and microlevel pandemic response teams bear responsibility for maintaining the provision of healthcare. The defaults of local public health authorities are decisive for the pandemic response team’s work. Main tasks and measures were the provision of information and the procurement and distribution of resources.

**Discussion:**

In terms of preparing for future pandemic situations, the knowledge gained will help to address concerns about maintaining healthcare for specific population groups, such as seriously ill and dying people, to the locally differing responsible bodies, even under pandemic conditions.

## Einleitung

Mit dem Ausbruch von COVID-19 (Coronavirus-Krankheit-2019) im Dezember 2019 ist das deutsche Gesundheitssystem durch die hohe Ausbreitungsgeschwindigkeit des SARS-CoV-2-Virus („severe acute respiratory syndrome coronavirus type 2“) sowie die regionale Variabilität und anhaltende Dynamik vor enorme Herausforderungen gestellt worden. Ein föderales politisch-administratives System leistet in Deutschland die Pandemiebewältigung [1]. Während die Entscheidungs- und Verantwortungsfunktionen bei politischen Handlungsträgern von Bund und Ländern (Makroebene) liegen, ist die Umsetzung dieser Entscheidungen die Aufgabe der Verwaltung der Länder und Kommunen (Mesoebene). Deren regionale Vorgaben sind wiederum maßgeblich u. a. für die Patientenversorgung in lokalen Gesundheitseinrichtungen und -diensten (Mikroebene). Zur Unterstützung der bestehenden Strukturen wurden im Rahmen der COVID-19-Pandemie auf allen Ebenen Krisenstäbe etabliert, die ein wichtiges Instrument im Krisenmanagement darstellen.

Ziel dieses Artikels ist es, die Zusammensetzung, Organisation und (Weisungs‑)Befugnisse von Pandemiekrisenstäben zu beschreiben, die während der Studie „PallPan“ zur Versorgung Schwerkranker, Sterbender und Angehöriger in Pandemiezeiten [2] exploriert wurden. Die Ergebnisse der „PallPan“-Studie mündeten in eine nationale Strategie für die Palliativversorgung in Pandemiezeiten [3]. Die Kenntnis struktureller Voraussetzungen im Pandemiemanagement ermöglicht es, die Interessen bestimmter Patientengruppen (z. B. Schwerkranker und Sterbender) gezielt an verantwortliche Stellen zu adressieren und Verbesserungen in der Versorgung zu initiieren. Welche Herausforderungen die Pandemie für die Versorgung von Schwerkranken, Sterbenden und ihren Angehörigen mit sich bringt und wie Krisenstäbe konkreten patientenbezogenen Auswirkungen der Pandemie begegneten, wurde ebenfalls erforscht [4].

## Methoden

In einem qualitativen Forschungsdesign wurden teilstrukturierte Interviews mit Vertreter:innen von Krisenstäben geführt, die mittels qualitativer Inhaltsanalyse ausgewertet wurden. Die Interviewten berichteten im Zeitraum Oktober 2020 bis Februar 2021 von den Tätigkeiten in Krisenstäben, wie sie vom Zeitpunkt der Einstufung von COVID-19 als Pandemie bis zum Winter 2020/2021 stattgefunden haben. Dieser Zeitraum wurde in 3 Phasen unterteilt, die sich an den Veränderungen der Inzidenzwerte im Pandemieverlauf orientieren, aber lokal zeitlich versetzt sein können (Tab. 1).

**Table 1:** 

Phase 1	Zeitraum zwischen der Ausrufung der COVID-19-Pandemie durch die Weltgesundheitsorganisation (WHO) und dem ersten Rückgang der Infektionszahlen (März bis etwa Juni 2020)
Phase 2	Zeitraum zwischen ersten Maßnahmenlockerungen und dem erneuten Anstieg der Infektionszahlen (etwa Juni bis September 2020)
Phase 3	Erneut steigende Inzidenzwerte (etwa ab Oktober 2020) bis zum Ende der Befragung im Februar 2021

### Rekrutierung

Aufgrund fehlender Übersichten zu Krisenstäben wurde eine Strategie mit unterschiedlichen Zugangswegen zu potenziellen Teilnehmenden angewandt. Die Kooperationspartner:innen im Projekt PallPan wurden um Hinweise gebeten, die beiden bevölkerungsreichsten Landkreise je Bundesland und Städteverwaltungen (> 100.000 Einwohner:innen) wurden schriftlich kontaktiert und eine Handsuche erfolgte im Internet (Suchbegriffe: Krisenstab, Task-Force, COVID-19 oder Corona, Krisenmanagement und spezifische Gesundheitseinrichtungen). Einschlusskriterien waren Volljährigkeit, Mitarbeit in einem Krisenstab mit Aufgabenbereich Gesundheitswesen (Mitglied oder beratend). Die Auswahl der Studienteilnehmenden erfolgte zielgerichtet, um möglichst unterschiedliche Krisenstäbe einzubinden (Inzidenz in der Region, Bundesland, beruflicher Hintergrund, Arbeitsplatz und Krisenstabebene).

### Datenerhebung

Die teilstrukturierten Interviews wurden per Video und in einem Fall telefonisch von Mitgliedern der Arbeitsgruppe durchgeführt, die im Vorfeld ein Interviewtraining absolviert hatten. Der Leitfaden enthielt Fragen zu folgenden Themen: Aufgaben des Krisenstabes, Berücksichtigung der Situation von Menschen am Lebensende und trauernder Angehöriger, Zusammensetzung, Organisation, Befugnisse und Kommunikationswege sowie personen-, berufs- und funktionsbezogene Fragen an die Teilnehmenden.

### Datenauswertung

Die Interviews wurden wörtlich transkribiert [5]. Mittels der Software MAXQDA 2020 (Verbi Software, Berlin, Deutschland, Release 20.04.0; [6]) wurde eine qualitative Inhaltsanalyse [7, 8] vorgenommen. Die Codierung erfolgte deduktiv mittels eines vorher festgelegten Codesystems, das anhand der Forschungsfrage und des Interviewleitfadens von der Arbeitsgruppe entwickelt wurde.

Um die Strukturen der Makroebene nachzuvollziehen, wurden begleitend Regierungserklärungen [9], Gesetze [10, 11], Beschlüsse [12, 13], Pressekonferenzen [14, 15] und Richtlinien [16] analysiert, da eine Befragung von Krisenstabsmitgliedern der Makroebene nicht stattfinden konnte (siehe Limitationen).

## Ergebnisse

### Studienteilnehmende und Datenerhebung

Von 120 Angefragten nahmen schließlich 41 Krisenstabsmitglieder teil. Eine weitere Person wurde in die Studie eingeschlossen, die über das Krisenmanagement auf Makroebene berichtete, jedoch nicht Mitglied eines Krisenstabes war. Die Krisenstabsmitglieder berichteten über 43 Krisenstäbe der Meso- und Mikroebene (20 der Meso- und 23 der Mikroebene). Die Teilnehmenden waren im Mittel 50 Jahre alt (33–68 Jahre, Standardabweichung SD 9,1), zu 66,7 % männlich und meist in Leitungspositionen mit Personalverantwortung (90,5 %). 69,1 % hatten mehr als 10 Jahre Berufserfahrung. Fast 2 Drittel der Befragten arbeiteten im Gesundheitswesen, ein Drittel im Katastrophenschutz. Die Interviews dauerten durchschnittlich 37 min (SD 15,24; Spanne 10–70 min).

### Strukturelle Besonderheiten im deutschen Krisenmanagement

**Figure 1:**
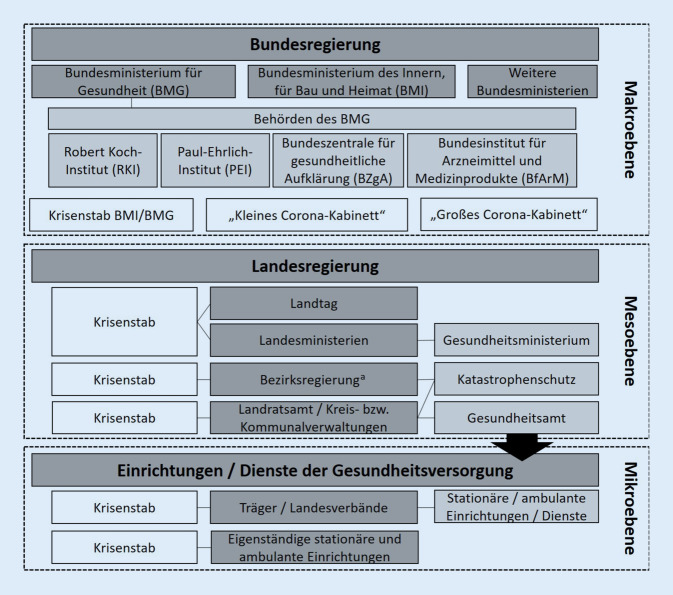

Eine Übersicht über die Hauptakteure im deutschen Pandemiekrisenmanagement auf Makro‑, Meso- und Mikroebene ist in Abb. 1 dargestellt.

#### Makroebene

Deutschland ist föderal organisiert und verwaltet [1]. Mit der Einstufung von COVID-19 als Pandemie am 11.03.2020 [17] rückte das nationale Infektionsschutzgesetz (IfSG) verstärkt in den Vordergrund, welches um den § 5 („Gesetz zum Schutz der Bevölkerung bei einer epidemischen Lage von nationaler Tragweite“) erweitert wurde [10]. Hiermit wird dem Bundesministerium für Gesundheit (BMG) die Befugnis zur bundeseinheitlichen Koordinierung der Coronamaßnahmen eingeräumt [9, 18, 19].

#### Mesoebene

Entsprechend der föderalen Struktur obliegt den Bundesländern die landeseigene Verwaltung [19]. Dabei tragen die obersten Landesbehörden die Verantwortung für die Umsetzung der Kabinettsbeschlüsse und spezifizieren diese in sog. Landesverordnungen. Diesbezüglich verantworten die Gesundheitsministerien der Länder die Informationsbereitstellung für die Öffentlichkeit und die Weitergabe von Empfehlungen. Auf allen Ebenen der Landesregierung wurden für die Zeit der Pandemie Krisenstäbe etabliert (Abb. 1), die v. a. für die Abstimmung zwischen den Behörden sowie für die Lageberichterstattung und Koordinierung notwendiger Maßnahmen verantwortlich sind.

Aus Interviews mit Vertreter:innen der Krisenstäbe auf Mesoebene geht hervor, dass v. a. Landkreise und kreisfreie Städte im Mittelpunkt der Krisenbewältigung stehen. Sie stehen in der Hauptverantwortung Maßnahmen zum Schutz der Gesundheit einzuleiten und umzusetzen. Dies ist auf das Prinzip der kommunalen Selbstverwaltung (Art. 28 Abs. 2 Grundgesetz) zurückzuführen. Regional sehr stark variierende Infektionszahlen können dadurch berücksichtigt werden. In vielen Städten und Landkreisen werden sog. Katastrophenschutzstäbe (Stab einer Katastrophenschutzbehörde) vorgehalten, die im Falle außergewöhnlicher Lagen, wie einer Pandemie, aktiviert werden können. Die Aktivierung dieser Katastrophenschutzstäbe und die Initiierung weiterer kommunaler Krisenstäbe obliegt den Hauptverwaltungsbeamt:innen (Landrat/Landrätin, (Ober‑)Bürgermeister:in). Die personelle Zusammenstellung der kommunalen Krisenstäbe erfolgte zumeist in Anlehnung an bestehende Katastrophenschutzkonzepte (z. B. Dienstvorschrift 100, Nordrhein-Westfalen), welche u. a. die Unterstützung durch den Katastrophen- und Bevölkerungsschutz oder die Berufsfeuerwehr vorsehen. Tab. 2 ist zu entnehmen, aus welchen Mitgliedern die jeweiligen Krisenstabsteams der Interviewteilnehmenden zusammengesetzt waren. Es obliegt den Hauptverwaltungsbeamt:innen über lokale Coronamaßnahmen zu entscheiden, die von den kommunalen Krisenstäben auf Grundlage aktueller Lageberichte vorbereitet werden. „Die Krisenstäbe müssen ihre Empfehlungen oder ihre Entscheidung gegenüber den jeweiligen Hauptverwaltungsbeamten rechtfertigen, sprich, einmal der Landkreiskrisenstab gegenüber dem Landrat, der Stadtkrisenstab gegenüber dem Oberbürgermeister“ (ID_F, Pos. 82)*.*

**Table 2:** 

Ebene	Feste Mitglieder	Bedarfsweise Mitglieder	Einrichtungsspezifische Mitglieder
Meso	Amstvertreter:innen der Bereiche Gesundheit, Ordnung, Recht, Personal; Vertreter:innen der Hygiene, Öffentlichkeitsarbeit, Feuerwehr, Polizei, Rettungsdienst, Katastrophenschutz	Vertreter:innen von Kliniken, Arztpraxen, Altenpflege, Bauamt, Kassenärztlicher Vereinigung, Pflege, Bildung, Kinderbetreuung, Medizinprodukteaufsicht, Impflogistik, Lebensmittelbeschaffung	(Ober‑)Bürgermeister:in; Vertreter:innen von IT-Amt, Sozial- und Jugendamt, Finanzamt, Amt für Flüchtlingsangelegenheiten, Kliniken, Infektionsschutz
Mikro	Geschäftsführer:in Pandemiebeauftragte	Vertreter:innen der Fachabteilungen, Personalrat	Vertreter:innen der Einrichtungen und verbandspezifischer Stabsstellen

Unter allen öffentlichen Verwaltungseinrichtungen kommt den Gesundheitsämtern eine tragende Rolle zu. Diese sind u. a. mit der Aufgabe der Hygieneüberwachung betraut und treffen regional verbindliche Einzelfallentscheidungen (z. B. Quarantäne, Hygienekonzepte, Kontaktverbote) in allen öffentlichen Bereichen (z. B. Gesundheitsversorgung, Gastronomie, Kinderbetreuung, Bildung). Gesundheitsämter sind dazu befugt, die Umsetzung verordneter Maßnahmen durch Vollzugsbehörden (Polizei, Ordnungsamt) zu kontrollieren. Dies ermächtigt kommunale Krisenstäbe, an denen Gesundheitsämter beteiligt sind, dazu, Allgemeinverfügungen zu erlassen. Auf dieser Grundlage treten Gesundheitsämter auch als Fachberatung in Katastrophenschutzbehörden und Leitung von Katastrophenstäben auf. Krisenstäbe, die innerhalb von Gesundheitsämtern etabliert wurden, haben die Aufgabe der Lagefeststellung und Berichterstattung, der Beratung und Unterstützung des Gesundheitsamtes und der Zuweisung von Aufgaben zu den entsprechenden Ämtern und Behörden (z. B. Ordnungsamt, Schulamt, Polizei). Gesundheitsämter stellen die zentrale Schnittstelle zwischen Meso- und Mikroebene dar.

#### Mikroebene

Zur Mikroebene zählen ambulante und stationäre Einrichtungen der Gesundheitsversorgung, unabhängig von deren Trägern (Abb. 1). Im Rahmen der durchgeführten Studie standen vor allem Einrichtungen mit palliativmedizinischem Versorgungsauftrag im Fokus der Untersuchungen, z. B. Krankenhäuser, Hospize, Alten- und Pflegeheime und ambulante Pflegedienste. In allen befragten Strukturen wurden für die Zeit der COVID-19-Pandemie Krisenstäbe zur Koordinierung einrichtungsinterner COVID-19-Maßnahmen etabliert. Sie wurden zumeist durch die Einrichtungsleitung bzw. den Träger, den Vorstand, die Klinikeinsatzleitung oder den Katastrophenschutzbeauftragten initiiert. „Also der Krisenstab existiert nicht immer, sondern der ist ins Leben gerufen worden im März mit Beginn der Pandemie und der Erkenntnis, dass es sozusagen einer Bündelung der Aktivitäten in Bezug auf die Pandemie braucht. Und der Initiator war in diesem Fall der Vorstand“ (ID_P, Pos. 31).

Die personelle Zusammenstellung der Krisenstabsteams und die Benennung einer Krisenstabsleitung obliegt den Initiator:innen und erfolgt in Anlehnung an bestehende Krisenpläne. Die Interviewteilnehmenden der Mikroebene berichteten, aus welchen Mitgliedern die jeweiligen Krisenstabsteams zusammengesetzt waren (Tab. 2).

Allen Krisenstäben auf Mikroebene ist die praktische Ausgestaltung der COVID-19-Maßnahmen entsprechend der Landesverordnung und kommunaler Rahmenvorgaben in Eigenverantwortung vorbehalten. Die Beschlüsse und Anweisungen des internen Krisenstabes sind für alle Mitarbeiter:innen der Einrichtung bindend. Die Beschlüsse von Krisenstäben überregionaler Träger haben Empfehlungscharakter und erfüllen vorrangig koordinierende und unterstützende Funktionen. Die Gesundheitsämter prüfen schriftliche Pandemiepläne (teilweise auch „Hygienekonzept“ genannt) und die Umsetzung der darin festgehaltenen Maßnahmen wird durch Amtsärzte kontrolliert.

### Aufgaben, Maßnahmen und Herausforderungen der Krisenmanagementstrukturen

#### Informationsbereitstellung

Die Krisenstabssitzungen der beiden Coronakabinette und des Regierungskabinetts (Makroebene) fanden ab Beginn der Pandemie wöchentlich statt [9]. Die resultierenden Regierungsbeschlüsse wurden in vierwöchigem Turnus auf den Internetseiten der Bundesregierung veröffentlicht. Auf Meso- und Mikroebene trafen sich Krisenstabsmitglieder zunächst täglich und informierten sich über die tagesaktuelle Infektionslage und die Beschlüsse seitens der Bundesregierung. In der zweiten Pandemiephase sank der Bedarf an regelmäßigen Treffen. Der Informationsaustausch innerhalb der Krisenstäbe sowie mit den Mitarbeitenden der Einrichtungen und Dienste erfolgte zumeist digital (z. B. Videokonferenz, Telefon, E‑Mail, Intranet, digitale Mitarbeiterzeitschrift), in einzelnen Fällen auch persönlich entsprechend den Hygienevorschriften.

Die Öffentlichkeit wurde z. B. über Homepages, Videobeiträge, Plakate, die Presse oder Rundschreiben informiert, auch wurden Möglichkeiten zur Rückmeldung aus der Öffentlichkeit geschaffen, darunter Bürgertelefone, elektronische Postfächer oder Onlineformulare. Einige wenige der befragten Krisenstäbe informierten auch Patient:innen und Angehörige über interne Newsletter und Flyer in den Eingangsbereichen der Einrichtungen.

Hinsichtlich der Kommunikation bemängeln Abgeordnete des Bundestages eine unzureichende Abstimmung und Koordinierung zwischen den Gesundheitsämtern sowie Rückstände bei deren Digitalisierung [19]. Zudem wird der Grad der Einflussnahme politischer Akteure in organisatorische Abläufe auf Meso- und Mikroebene i. A. als zu gering empfunden. Auf Meso- und Mikroebene bestanden insbesondere kurz nach Einstufung von COVID-19 als Pandemie v. a. Unklarheiten hinsichtlich des Infektionsgeschehens, der Ansteckungswege sowie über adäquate Schutzmaßnahmen. Vertreter:innen der Krisenstäbe auf Mesoebene berichteten, dass sich die überregionalen Vorgaben (Richtlinien, Verordnungen, Verfügungen) übergeordneter Behörden fortlaufend geändert hätten. Die Kommunikation hierzu wurde als unklar und zufallsgesteuert, die Anzahl der Empfänger als groß, vielfältig und teilweise unübersichtlich beschrieben. Eine passgenaue Auswahl oder Bündelung von Informationen für bestimmte Adressatengruppen war zunächst aufgrund der Dichte, Dynamik und des Zeitmangels nicht möglich. „Ja, der Krisenstab muss einerseits die Lage auch in ihrer Dynamik, also insofern engmaschig dann erfassen. Also die Lage ist sowohl … die Zahlen von Erkrankten, Erkrankungsverdächtigen, aber dann halt auch von den … sich auch ständig ändernden überregionalen Vorgaben an Richtlinien, Verordnungen, Verfügungen. Muss das sichten und muss dann die Beteiligten, insbesondere die Fachöffentlichkeit, aber auch die breite Öffentlichkeit dann informieren und ja, Prozeduren dann anpassen und auch wieder zurücknehmen. Muss die Lage dann auch monitoren, wo halt Bedarf besteht und muss dann selektiv nachsteuern“ (ID_W, Pos. 21).

Da Mitarbeitende den Wunsch nach schnellen Entscheidungen äußerten, fühlten sich Krisenstabs- bzw. Einrichtungsleitungen dazu gedrängt, umgehend Entscheidungen zu treffen, ohne nach ihrem Empfinden ausreichende Informationsgrundlagen zu haben. Das Ausbleiben schriftlicher Antworten von Gesundheitsamt oder Heimaufsicht sorgte bei Einrichtungsvertreter:innen zusätzlich für Unklarheiten bezüglich der Verantwortlichkeit.

Auf allen Ebenen wurde von Bemühungen berichtet, den unklaren und komplexen Kommunikationswegen und der empfundenen Flut an Informationen entgegenzuwirken. Informationen wurden an zentralen Stellen gesammelt, strukturiert und zumeist in Form von Handlungsempfehlungen weitergegeben. Abgestimmte Anregungen, Empfehlungen und Nachfragen von Gesundheitseinrichtungen und öffentlichen Ämtern wurden gebündelt an verantwortliche Stellen rückgemeldet. Fehlinformationen im Internet wurden als herausfordernd wahrgenommen. „Dann kamen auch Berichte über YouTube. Es gibt immer wieder so Leute, die dann querschießen, ohne dass das wissenschaftlich fundiert ist. Und dann habe ich auch dem Geschäftsführer gesagt: Lassen Sie uns bei den Fakten bleiben und lassen Sie uns bei dem bleiben, was die Experten sagen, und nicht, was irgendjemand daher sagt und ins Netz streut“ (ID_B, Pos. 26).

#### Beschaffung und Verteilung von Ressourcen

Zu Beginn der Pandemie wurde von den zuständigen Mitarbeiter:innen der Krisenmanagementstrukturen auf Makroebene eine COVID-19-spezifische Ergänzung zum nationalen Pandemieplan (Stand 04.03.2020) erarbeitet [20]. Weiterhin nahm die Bundesregierung Anpassungen an der Gesetzgebung (IfSG) vor und stellte finanzielle Mittel für die Pandemiebewältigung im Bereich der Gesundheitsversorgung, u. a. die Beschaffung von Schutzmaterialien, den Ausbau von Testkapazitäten und Intensivstationen, zur Verfügung [19, 21]. Zudem richtete die Bundesregierung einen finanziellen Schutzschirm für Krankenhäuser und niedergelassene Ärzt:innen ein, um so Einnahmeausfälle und erhöhte Kosten abzufedern [21].

Während der Zweck der finanziellen Mittel durch die Bundesregierung vorbestimmt wird, verantworten die untergeordneten Behörden deren konkreten Einsatz. Interviewte auf Mesoebene berichteten von Engpässen bei persönlicher Schutzausrüstung sowie von Bemühungen um deren Beschaffung und Verteilungsgerechtigkeit. „Also vor allem anfangs ging es ja um relativ geringe Fallzahlen, da ging es eher um Schutzausrüstung und Materialvorräte und prophylaktische Sicherstellung einer korrekten Arbeitsweise und im Weiteren ging es dann eher um das Monitoring von Behandlungskapazitäten gerade im stationären Bereich“ (ID_W, Pos. 27)*.* Weitere Maßnahmen galten der Bereitstellung von ausreichend Intensivkapazitäten, der Aufrüstung von Rettungswägen mit Beatmungsgeräten, räumlichen Baumaßnahmen (z. B. Einrichtung von COVID-Stationen) und vereinzelt auch Vorbereitungen der nötigen Infrastruktur für eine große Anzahl infizierter Toter.

Krisenstäbe der Mikroebene entwarfen zunächst Pandemiepläne bzw. Hygienekonzepte oder passten bestehende Pläne an die jeweils aktuellsten Allgemeinverfügungen fortlaufend an. Während auch auf Mikroebene v. a. in der ersten Pandemiephase Schutzmaterial fehlte, stellten sich zusätzlich Fragen zur korrekten Anwendung der Hygienemaßnahmen. Zudem warf die Materialbeschaffung nicht nur logistische, sondern auch finanzielle Fragen auf. Um den Ressourcenengpässen zu begegnen, wurden verschiedene Maßnahmen ergriffen: Da Hersteller Anfragen nach geringen Stückzahlen nicht berücksichtigten, schlossen sich mehrere Gesundheitseinrichtungen und Träger zur Materialbeschaffung zusammen, um in besserer Verhandlungsposition zu sein. Große Einrichtungen wie Krankenhäuser etablierten Systeme, die den aktuellen Lagerbestand monitoren und Hochrechnungen des Bedarfs an Hygienematerial ermöglichen. Mit Anpassungen der IT-Infrastruktur wurden teilweise auch Meldesysteme von Infektions- bzw. Verdachtsfällen und Ausbrüchen eingerichtet. Die Forderung, neue Intensivkapazitäten zu schaffen, stellte die Krisenstäbe laut Aussagen der Befragten vor enorme Herausforderungen. Andere Baumaßnahmen, wie der Bau eines neuen Hospizes, mussten unterbrochen werden. Zudem wurde die Organisation technischer Voraussetzungen für das Homeoffice von Verwaltungspersonal notwendig.

#### Mitarbeiterbelastung

Interviewte von Gesundheitsämtern berichteten, dass sie über die hohe Belastung des Pflegepersonals auf Mikroebene informiert wurden. Gründe bestünden v. a. im Personalmangel, insbesondere in der Fachpflege für Intensivmedizin, im Zeitdruck sowie in der Umsetzung neuer Hygieneregeln und Aufgaben. Um der Aufgabe der Kontaktpersonennachverfolgung nachgehen zu können, stockten Gesundheitsämter Personal auf, was jedoch nicht ausreichte um nichtpandemiebezogenen Aufgaben der öffentlichen Gesundheitssorge nachzukommen. Zusätzlich belastet waren Mitarbeitende in Gesundheitsämtern aufgrund zahlreicher Beschwerden und des zunehmenden Drucks aus der Öffentlichkeit.

Vertreter:innen der Einrichtungen und deren Träger berichteten, dass der ohnehin bestehende Mangel an Personal durch COVID-19-Erkrankungen, Quarantänemaßnahmen, hohe Belastung oder Einholung der geringen Ausfallzeiten in der ersten Pandemiephase verstärkt wurden. Zusätzliche Belastung brächten Mehraufgaben, die durch Administration, Hygienemaßnahmen und die Auseinandersetzung mit Angehörigen entstünden. Die Qualifikation zur Anwendung von Hygienemaßnahmen bei ungelerntem Personal wurde teilweise als Herausforderung beschrieben, aber auch die Entwicklung von Konzepten zur Mitarbeiterqualifikation bei kurzfristigem Einsatz in anderen Kliniken. Personalengpässen wurde entgegengewirkt durch Umverteilung von Aufgaben, längere Arbeitszeiten, Hinzuziehung von Personal aus anderen Einrichtungen desselben Trägers oder Einbezug von Verwaltungspersonal in die Pflege, Schließung von Stationen, Anpassung der Quarantäne für Personal via Gesundheitsamt und Konzepte zur Testung von Mitarbeitenden.

Für den Schutz des Personals wurden vielerorts Maßnahmen ergriffen, um sie vor Ansteckung und Übertragung zu schützen: Reduktion der physischen und vermehrt telefonische Patientenkontakte, Umstellung auf Homeoffice und entsprechende Anschaffung technischer Ausrüstung, abwechselnde Büroanwesenheitszeiten, Trennung von Räumlichkeiten, Pausenregelungen, Schichteinteilung, Regelung zur Nutzung von Dienstwägen, Dienstreiseverbote.

Die Situation hatte Auswirkungen auf das Wohlbefinden des Personals. Unterschiedliche Haltungen der Mitarbeiter:innen (Angst, Gewissenhaftigkeit, Strenge, Wohlwollen) führten zu Unsicherheiten und Konflikten. Alternativen für Austausch und soziales Zusammensein der Mitarbeitenden wurden deshalb geschaffen, wie beispielsweise eine Onlineweihnachtsfeier oder eine Hotline. Ein hoher Druck aus der Öffentlichkeit und Schuldzuweisungen bei Ausbrüchen erschwerten die Situation zusätzlich, wie auch persönliche pandemiebedingte Aspekte wie eine fehlende Kinderbetreuung. In einigen Einrichtungen wurde für Mitarbeitende eine Kinderbetreuung organisiert. Auch der Umgang mit der Trauer der Mitarbeitenden angesichts vieler Todesfälle in den Einrichtungen und auch im privaten Umfeld wurde von Krisenstabsmitgliedern als ein wichtiges Thema, vor allem ab der zweiten Phase beschrieben. Einzelne Krisenstäbe entwickelten Handlungsempfehlungen zum Abschiednehmen für die Mitarbeitenden.

Als eine weitere Problematik beschrieben Krisenstabsmitglieder die eingeschränkten Aus‑, Fort- und Weiterbildungen der Ehrenamtlichen sowie das Fehlen von Supervisionen und des Austauschs zwischen den aktiven Ehrenamtlichen. Als Maßnahmen wurden Versuche unternommen, den Einsatz der ehrenamtlichen Hospizbegleiter:innen wieder zu ermöglichen.

## Diskussion

Während national häufig scharfe Kritik an politischen Entscheidungen und der Uneinheitlichkeit zwischen den Ländern geäußert wurde [22], hob die ausländische Presse hervor, dass Deutschland bei der Eindämmung der Coronapandemie effektiver als andere Nationen war [23, 24]. Wenngleich notwendige Voraussetzungen zur Pandemiebewältigung (z. B. Anpassungen des IfSG, Bereitstellung finanzieller Mittel und Beschluss von Lockdowns) von zentraler Stelle geschaffen werden, so liegt die Hauptverantwortung für den Schutz der Gesundheit bei den Kommunalverwaltungen und den Einrichtungen selbst [25, 26]. Insbesondere die rund 400 lokalen Gesundheitsämter haben ein sehr breites Aufgabenspektrum [27]. Sie organisieren sich in Eigenverantwortung und treffen Entscheidungen entsprechend des IfSG nach eigenem Ermessen.

Die Krisenstäbe auf Makro‑, Meso- und Mikroebene nehmen eine zentrale Rolle im Pandemiemanagement ein und werden in Akutsituationen temporär aktiviert bzw. initiiert. Für die personelle Zusammenstellung der Krisenstabsteams sind die Pandemiekrisenstäbe selbst verantwortlich [28, 29]. Hinsichtlich der vertretenen Fachdisziplinen sowie der hierarchischen Position der Mitglieder sind die Krisenstäbe sehr heterogen besetzt [30]. Diese reichen von hochrangigen Führungspositionen über beratende Fachvertreter:innen bis hin zu Unterstützungspersonal [31]. Welche Entscheidungen Krisenstäbe treffen dürfen und wer die Beschlüsse verantwortet, ist davon abhängig, welche Aufgaben der jeweilige Krisenstab erfüllt und ob befugte Personen (z. B. Oberbürgermeister:innen, Amtsärzte/Amtsärztinnen) am Pandemiekrisenstab beteiligt sind [28]. Die Entscheidungsbefugnis obliegt zumeist den Initiatoren oder den benannten Verantwortlichen, wobei keine klaren Regelungen hinsichtlich der Beschlussfassung von Entscheidungen bekannt sind [28]. In Abhängigkeit von der zu lösenden Problemstellung handelt es sich zumeist um Einzelfallentscheidungen.

Das Aufgabenspektrum der Pandemiekrisenstäbe ist sehr vielfältig. Sie befassen sich mit der kurzfristigen Umsetzung von Maßnahmen und unterstützen insbesondere bei der Abstimmung zwischen den am Pandemiemanagement beteiligten Organisationsformen. Die dargestellten Herausforderungen hinsichtlich der Aufgabenbereiche Informationsbereitstellung, Ressourcenbeschaffung und Mitarbeiterbelastung zeigen jedoch, dass vor allem die Krisenstäbe der Mikroebene mangels erforderlicher Fähigkeiten und Hilfsmittel (z. B. fehlende Digitalisierung, unzureichende Daten- bzw. Informationsgrundlage, unzureichende Dokumentation und Kommunikation, fehlende Mitarbeiterkompetenzen, eingeschränkter Marktzugang) in ihrer Handlungsfähigkeit eingeschränkt sind [25, 30]. Die Bemühungen der Krisenstäbe, Informationen, die sie selbst von höheren Ebenen erhalten, zu bündeln und zielgruppengerecht bereitzustellen und die verschiedenen Adressaten (Patient:innen, Angehörige, eigene Mitarbeitende, Öffentlichkeit) zeitgerecht mit den für sie relevanten Informationen zu versorgen, kosten großen zeitlichen und personellen Aufwand. Strukturierte und transparente Informationswege können dabei helfen, die Informationsbereitstellung effizienter zu gestalten. Neben der Materialbeschaffung, die mit zeitlicher Verzögerung erfolgreich organisiert werden konnte, bleibt der Personalmangel einer der limitierenden Faktoren bezüglich der Aufgaben der Beschaffung und Verteilung von Ressourcen und ist ausschlaggebend für Belastungen der Mitarbeitenden.

Angesichts der Neuartigkeit und langen Dauer der Pandemie mussten Krisenstrukturen zunächst entwickelt werden. Im Forschungsprojekt egePan Unimed (www.egepan.de) wird ein Rahmenkonzept entwickelt, wie das regionale und föderale Pandemiemanagement optimiert werden kann. Im Sinne einer Vorbereitung auf mögliche zukünftige Pandemien können die vorliegenden Erkenntnisse die Entwicklung von Handlungsempfehlungen unterstützen, wie bereits im Rahmen des PallPan-Projektes erfolgt [3]. Interessenvertretungen für andere Patientengruppen könnten in ähnlicher Weise die vorliegenden Ausführungen nutzen.

## Limitationen

Zielgruppe der vorliegenden Studie waren Krisenstäbe von Einrichtungen mit palliativmedizinischem Versorgungsauftrag (Mikroebene) sowie die jeweils übergeordneten Krisenstäbe (Meso- und Makroebene). Krisenstäbe mit anderen Zuständigkeitsbereichen wurden nicht berücksichtigt. Bei den Studienteilnehmer:innen handelt es sich um eine nichtzufällige Stichprobe, doch erfolgte die Rekrutierung unter Einbezug verschiedener Perspektiven (Ebene, Region, Bevölkerungsdichte, Infektionslage, Setting). Auf nationaler Ebene stand innerhalb des kurzen Zeitrahmens der Studie kein Krisenstabsmitglied für ein Interview zur Verfügung, sodass Angaben zum Krisenmanagement auf Makroebene von einem Interviewteilnehmer aufgenommen und durch die Analyse aktueller Literatur ergänzt wurden. Die Anzahl Teilnehmender aus ländlichen Gebieten war gering. Angefragte Stellen und Einrichtungen in Kommunen hatten oft keine Krisenstäbe eingerichtet. Die vorliegenden Studienergebnisse beruhen auf exemplarischen Berichten, deren Verallgemeinerbarkeit zwar begrenzt ist, die jedoch die strukturelle und organisatorische Vielfalt bestehender Pandemiekrisenstäbe gut darstellen.

## Schlussfolgerung

Das Pandemiemanagement in Deutschland steht den Herausforderungen eines föderalen Organisationsprinzips sowie regional sehr unterschiedlichen Infektionslagen, Versorgungs- und Entscheidungsstrukturen gegenüber. Während Bund und Länder bundeseinheitliche Rahmenbedingungen schaffen, liegt die Hauptverantwortung für den Schutz der Gesundheit zumeist bei den einzelnen Einrichtungen selbst. Dabei unterstützen Pandemiekrisenstäbe die kurzfristige Umsetzung lokaler Coronamaßnahmen sowie die Koordination zwischen beteiligten Behörden und Organisationen. Einheitliche Regelungen hinsichtlich der Initiierung, personellen Zusammenstellung, Aufgaben, Zuständigkeiten und Befugnisse von Krisenstäben gibt es jedoch nicht. Zudem fehlt eine Übersicht aktiver Pandemiekrisenstäbe. Interessenvertretungen für spezifische Patientengruppen wie Schwerkranke und Sterbende sind deshalb gut beraten sich über die Strukturen und Herausforderungen des Pandemiemanagements insgesamt zu informieren, um ihre Anliegen an die sich lokal unterscheidenden verantwortlichen Stellen vor allem auf den Ebenen der Patientenversorgung und der öffentlichen Verwaltung wirkungsvoll anzubringen.
